# Using population attributable risk to choose HIV prevention strategies in men who have sex with men

**DOI:** 10.1186/1471-2458-11-247

**Published:** 2011-04-19

**Authors:** Rebecca J Guy, Handan Wand, David P Wilson, Garrett Prestage, Fengyi Jin, David J Templeton, Basil Donovan, Andrew E Grulich, John M Kaldor

**Affiliations:** 1 Kirby Institute (formerly the National Centre in HIV Epidemiology and Clinical Research), Sydney, NSW, Australia; 2 Royal Prince Alfred Hospital, Sydney South West Area Health Service, New South Wales, Australia; 3 Sydney Sexual Health Centre, Sydney Hospital, Sydney, New South Wales, Australia

## Abstract

**Background:**

In Australia, HIV is concentrated in men who have sex with men (MSM) and rates have increased steadily over the past ten years. Health promotion strategies should ideally be informed by an understanding of both the prevalence of the factors being modified, as well as the size of the risk that they confer. We undertook an analysis of the potential population impact and cost saving that would likely result from modifying key HIV risk factors among men who have sex with men (MSM) in Sydney, Australia.

**Methods:**

Proportional hazard analyses were used to examine the association between sexual behaviours in the last six months and sexually transmissible infections on HIV incidence in a cohort of 1426 HIV-negative MSM who were recruited primarily from community-based sources between 2001 and 2004 and followed to mid-2007. We then estimated the proportion of HIV infections that would be prevented if specific factors were no longer present in the population, using a population attributable risk (PAR) method which controls for confounding among factors. We also calculated the average lifetime healthcare costs incurred by the HIV infections associated with specific factors by estimating costs associated with clinical care and treatment following infection and discounting at 3% (1% and 5% sensitivity) to present value.

**Results:**

Unprotected anal intercourse (UAI) with a known HIV-positive partner was reported by 5% of men, the hazard ratio (HR) was 16.1 (95%CI:6.4-40.5), the PAR was 34% (95%CI:24-44%) and the average lifetime HIV-related healthcare costs attributable to UAI with HIV-positive partners were $AUD102 million (uncertainty range: $93-114 m). UAI with unknown HIV status partners was reported by 25% of men, the HR was 4.4 (95%CI:1.8-11.2), the PAR was 33% (95%CI:26-42%) and the lifetime incurred costs were $AUD99 million. Anal warts prevalence was 4%, the HR was 5.2 (95%CI:2.4-11.2), the PAR was 13% (95%CI:9-19%) and the lifetime incurred costs were $AUD39 million.

**Conclusions:**

Our analysis has found that although UAI with an HIV-positive sexual partner is a relatively low-prevalence behaviour (reported by 5% of men), if this behaviour was not present in the population, the number of infections would be reduced by one third. No other single behaviour or sexually transmissible infections contributes to a greater proportion of infections and HIV-related healthcare costs.

## Background

In Australia, there were 1050 new cases of HIV diagnosed in 2009, bringing the estimated number of people living with HIV infection by the end of that year to 20,171[1]. HIV has been highly concentrated among men who have sex with men (MSM) since the epidemic began nearly 30 years ago. After a long decline, rates of HIV diagnosis in MSM began increasing ten years ago and have continued to do so, almost certainly reflecting a resurgence in incidence of infection [2]. Unprotected anal intercourse (UAI) has been identified as the main mode of HIV acquisition in Australia [3,4] and the frequency of this behaviour has increased steadily since the mid-1990s [5,6].

The broad category of UAI in fact represents a spectrum of behaviours, which have documented different levels of HIV risk [7] and are also perceived by MSM as being associated with different levels of risk. This perception has led HIV-negative men wishing to engage in UAI to adopt behaviours that they believe reduce their risk of infection, including choosing partners perceived to be HIV-negative ("serosorting"), forming long term relationships involving explicit sexual agreements with partners who are HIV-negative ('negotiated safety), performing insertive anal intercourse only ("strategic positioning") and avoidance of ejaculation inside the rectum as the receptive partner ("withdrawal") [8].

Health promotion strategies for MSM have also recognised the need to make distinctions among these different subcategories of UAI, but have been complicated by ongoing debates as to which, if any of the modes can be recommended as "safer", let alone "safe". Until recently, there has also been a relative absence of quantitative data on the risk associated with the various forms of UAI, so that it has been difficult to undertake health promotion that is truly evidence based. Furthermore, biomedical prevention strategies such as circumcision and STI treatment are on the prevention agenda, without a comprehensive assessment of what population impact they could potentially achieve. In Australia, circumcision has been associated with a significant reduction in HIV incidence among those MSM who reported a preference for the insertive role in anal intercourse [9]. The early detection and treatment of curable anal and urethral sexually transmitted infections (STIs) has been suggested as a possible strategy for HIV prevention in MSM [10] based on observational studies demonstrating independent associations between HIV seroconverison and prior STIs in MSM [11,12].

Analyses of data from a large cohort of gay men has gone some way towards filling this gap in the evidence base for risk factors, by estimating the relative risk of HIV acquisition associated with specific subcategories of UAI, and other prevention related factors such as STI control and circumcision [8].

In this paper, we take this analysis further, through the use of the population attributable risk (PAR), which takes account of both the relative risk (RR) of specific risk factors, and their prevalence in the population. The PAR provides a quantitative assessment of the potential impact of risk factor on disease incidence in the population [13]. Instead of using the traditional method of calculating PAR, we use a more comprehensive PAR method described by Spiegelman (2007) [13] and Wand (2009) [14] which adjusts for the effects of other variables. The only PAR papers previously published in this field did not use this adjustment [15-17]. We also examine the estimated savings in HIV-related health care costs associated with each risk factor by estimating subsequent costs associated with clinical care and management of HIV infection.

## Methods

### Study Population

The study population consisted of men participating in the Health in Men (HIM) study, which was a prospective cohort of MSM in Sydney, Australia. HIV-negative men (*n *= 1426) who were non-randomly recruited primarily from community-based sources between 2001 and 2004 and followed to mid-2007 [8,18]. Participants underwent annual HIV testing, and detailed information on sexual risk behaviour was collected every 6 months.

### Sexual risk behaviours

We adopted definitions of sexual behaviours and partner choice strategies as in the earlier analysis by Jin and colleagues [8] and included strategic positioning, withdrawal and serosorting, as defined above. The definitions of risk reduction behaviours were based on exclusive practice. For example, if a man reported insertive UAI and any receptive UAI during a 6-month period, he was classified as not reporting strategic positioning in the period. The extent of practising each behaviour was quantified for all men at each six monthly cohort study interview.

We did not include substance use as a category in the model, as drugs used specifically to enhance sexual pleasure, particularly oral erectile dysfunction medications, have been associated with increased sexual risk behaviour, but are not direct risk factors for HIV transmission [19] and injecting drug use is not a major risk for HIV transmission in MSM in Australia [1].

### Circumcision

We included circumcision status as described in a paper by Templeton and colleagues [9]. Circumcision status was reported at baseline and self-reported circumcision status was validated by clinical examination in a subgroup of 240 consecutively presenting participants [20].

### STIs

We also included specific STIs in the PAR analysis selected on the basis of being found to be associated with a significant increased risk of HIV seroconversion in an earlier analysis by Jin and colleagues which adjusted for sexual risk behaviour [21]. These STIs included self-reported anal warts between cohort study visits and anal gonorrhoea at the cohort study visit. Infectious syphilis and herpes simplex virus 2 (HSV2) were not found to be significantly associated with HIV seroconversion in this analysis and were not included in our PAR regression model.

### HIV incidence

For HIV incidence, total person-years were calculated as the time from study entry to the estimated date of seroconversion, or to the end of the study in June 2007 for those who remained HIV-negative. The midpoint between interviews was used as the date of infection for participants who had an interval or study‐visit diagnosis. Identifiers were matched against the Australian national HIV register each year to identify infections which occurred in those who tested outside the study or had been lost to active follow-up.

### Population attributable risk

We adapted the method described by Spiegelman (2007) [13] and Wand (2009) [14] to estimate the PAR. PAR quantifies the potential impact of risk factor on disease incidence in the population. The PAR is calculated based on the RR or hazard ratio HR of the association between the risk factor (sexual behaviour or STI) and the outcome (HIV incidence), combined with the prevalence of the risk factor.

Specifically, the PARs were calculated as follows. When there is only one risk factor, at two levels (1 versus 0)(1)

Where *HR *is the hazard ratio, *p *is the prevalence of the risk factor in the population and *s *indexes the two strata determined by the value of the risk factor. Equation 1 can be generalized to the multi-factorial setting when there are more than one risk factors at multiple levels, as(2)

Where *HR*_*s *_and *p*_*s*_, *s *= 1,...*S*, are the hazard ratios and the prevalences in the target population for the *s *th combination of the risk factors. Full PAR can be estimated by using Equation 2 and interpreted as the percent reduction expected in the number of HIV seroconversion if all the known risk factors were eliminated from the target population.

In a multifactorial disease setting, at least some key risk factors such as age and sex are not modifiable. This limits the practical utility of the full PAR which is based on modification of all variables of interests. In an evaluation of a preventive intervention in a multifactorial disease setting, the interest is in the percent of cases associated with the exposures to be modified, when other risk factors, particularly non-modifiable ones are present but do not change as a result of the intervention. Therefore we derived and used partial PAR, assuming that the unmodifiable variable(s) remained unchanged.

Under the assumption of no interaction between the modifiable and non-modifiable risk factors of interest, the partial PAR is formulated as(3)

where *t *denotes a stratum of unique combinations of levels of all background risk factors which are not modifiable and/or not under study, *t *= 1,...,*T *and *HR*_2__*t *_is the hazard ratio in combination *t *relative to the lowest risk level, where *HR*_2,1 _= 1. As previously, *s *indicates a risk factor defined by each of the unique combinations of the levels of the modifiable risk factors, that is, those risk factors to which the PAR applies, *s *= 1,...,*S*, and *HR*_1__*s *_is the relative risk corresponding to combinations relative to the lowest risk combination, *HR*_1,1 _= 1. The joint prevalence of exposure group *s *and stratum *t *is denoted by *p*_*st*_, and . The PAR represents the difference between the number of cases expected in the original cohort and the number of cases expected if all subsets of the cohort who were originally exposed to the modifiable risk factor(s) had eliminated their exposure(s) so that their relative risk compared to the unexposed was 1, divided by the number of cases expected in the original cohort.

The HR for each of the sexual behaviours were determined using a Cox regression model. The prevalence of the behaviours were time dependent, taking account of behaviour each six months during the cohort. In our study, a univariate PAR analysis was undertaken, followed by a multivariate analysis which adjusts for the effects of other variables and assumes non-modifiable risk factors are unchanged. The PAR represents an estimate of the proportion of infections eliminated, taking account of relationships with other variables. We established two models. Model 1 included all factors with sexual behaviour broken down according to HIV status of the sexual partner. Model 2 included all factors with sexual behaviour broken down according to the sexual position. We were unable to include both sexual position and partner's serostatus in the same model because of the sparse data which led to empty cells in the combination levels.

95% CIs were estimated for individual risk factors using SAS statistical software, version 9 [22].

### HIV costs

**Figure 1:**
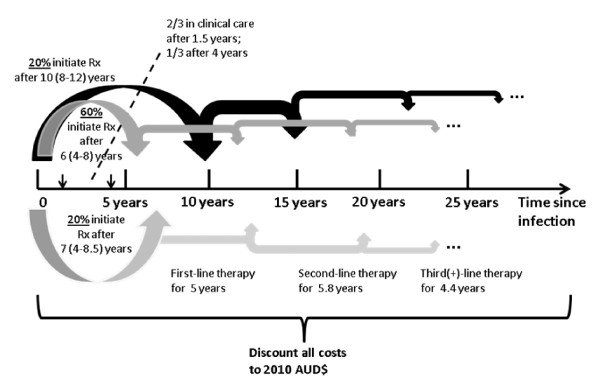
**Diagram describing assumptions of time delays incorporated in costing calculations**.

We estimated the average lifetime healthcare costs associated with each HIV infection (over 40 years post-infection), factoring the expected delays between infection and clinical care, including initiation of antiretroviral therapy, and discounting all costs to 2010 Australian dollars. Firstly, we estimated the average time from infection to initiation of treatment. Approximately 20% of people diagnosed with HIV in Australia have a CD4 count less than 200 cells per μl and a further 20% have a CD4 count between 200 and 350 cells per μl; the time between infection and diagnosis is assumed to be 10 years (uncertainty range: 8-12 years) and 7 years (4-8.5 years), respectively, based on average rates of CD4 decline (e.g. see [23]). This 40% of HIV-infected people are assumed to initiate therapy at the time of HIV diagnosis. A further 20% of HIV diagnoses in Australia have a CD4 count between 350 and 500 cells per μl at diagnosis and it is assumed that they have been infected for ~4 years and they will remain off antiretroviral medications for a further 2 (0-4) years. The remaining 40% of HIV diagnoses in Australia have a CD4 count greater than 500 cells per μl at diagnosis and it is assumed that they have been infected for ~1.5 years on average and they will remain off antiretroviral medications for a further 4.5 (2.5-6.5) years (half of this time with CD4 count greater than 500). The average annual healthcare costs for people living with diagnosed HIV but not yet receiving antiretroviral therapy is estimated to be AUD$1523 for people with a CD4 count greater than 500 and AUD$2055 for people with a CD4 count between 350 and 500, as published in the Australian 2009 Return on Investment 2 (ROI2) Report [24]. As previously estimated [24], standard first-line antiretroviral therapies in Australia cost AUD$14,613 per year, second-line therapies cost AUD$15,178 per year and third- and subsequent lines of therapy cost an average of AUD$27,776 per year. There is an estimated AUD$2731 in additional annual healthcare costs for people on therapy, associated with CD4 and viral load tests, general and specialist consultations, hospitalisations etc [24]. Based on antiretroviral pathway data from the clinic-based Australian HIV Observational Database (AHOD) [25], the mean average duration remaining on first-line regimens in Australia before switching to a second-line therapy is 5.0 years, and the average durations remaining on second- and subsequent-line of therapies are 5.8 years and 4.4 years, respectively. These assumptions around time delays between infection and initiating clinical care and treatment, when healthcare costs become relevant, are presented in Figure 1. We calculated lifetime costs associated with HIV infection, assumed to be over 40 years from the time of infection, discounting costs at a rate of 3% per year with sensitivity analyses of 1% and 5%. Accordingly, the estimated average lifetime HIV-related healthcare costs are AUD$429,662 ($391,107-477,626) for 3% discounting, and AUD$681,381 ($632,453-739,807) and AUD$282,267 ($251,419-322,219) for 1% and 5% discounting, respectively.

In recent years the numbers of HIV diagnoses in Australia have remained relatively stable at approximately 1000 cases diagnosed per year (annual average of 1027 over the past four years). Of these cases, 70.05% (~700 per year) were associated with men who reported sex with another men as the risk exposure [1]. To calculate the average healthcare costs incurred from HIV seroconversions associated with specific risk factors, we multiplied the PAR percent for each specific risk factor, by the 700 HIV infections associated with male homosexual exposure, by the average healthcare costs per HIV-infected person. We performed sensitivity analyses by rate of discounting and according to the bounds in delays in durations of time between infection and diagnoses and commencement of antiretroviral treatment but calculations were based on the best estimated PAR of the risk factors and not the 95% CI of the PAR.

## Results

### Prevalence of risk factors

Table 1 provides the frequency distributions of sexual behaviours and select STIs reported in the HIM study considered in this study.

**Table 1:** Prevalence of risk factors and association between these risk factors and HIV seroconversion (hazard ratio)

	**Prevalence of behavior n (%)**^**(1)**^	Number of HIV cases (person-years)	Hazard ratio (95% CI)
			

No UAI	521 (37)	7 (2071.4)	1

**UAI by reported HIV status of sexual partners**^(2)^			

UAI with HIV-negative only (serosorting)	466 (33)	14 (1958.7)	2.17 (0.88,5.39)

UAI with some unknown HIV status	362 (25)	13 (879.8)	4.44 (1.77,11.16)

UAI with any HIV-positive	77 (5)	13 (246.3)	16.12 (6.42,40.46)

**UAI by sexual position**^(2)^			

Insertive UAI only (strategic positioning)	225 (16)	4 (792.1)	1.54 (0.45,5.26)

Receptive with withdrawal (withdrawal)	204 (14)	11 (662.0)	5.00 (1.94,12.92)

Receptive with ejaculation	426 (33)	25 (1627.2)	4.65 (2.01,10.76)

**Circumcision status**^(3)^			

Uncircumcised men	488 (34)	17 (16.7)	1.22 (0.67,2.22)

**Number of sexual partners**			

10+ casual sexual partners in the last 6 m	528 (37%)	20 (1370.8)	2.1 (1.12-3.74)

**STIs**^(4)^			

Anal warts between study visit	59 (4)	8 (197.1)	5.22 (2.44,11.18),

Anal gonorrhoea at study visit	4 (<1)	3 (42.8)	8.50 (2.60,27.95)

CI = Confidence interval, UAI = Unprotected anal intercourse, m = months

1. Reported at baseline cohort asssessment

2. Adapted from Jin et al [8]

3. Adapted from Templeton et al [9]

4. Adapted from Jin et al [21]

#### UAI by reported HIV status of sexual partners

A third of men (33%) reported UAI with HIV-negative partners only, a quarter (25%) reported UAI with men of unknown status and 5% reported UAI with HIV-positive men.

#### UAI by sexual position

A third (33%) of the men reported receptive UAI with ejaculation, 14% reported receptive UAI with withdrawal and 16% insertive UAI only (strategic positioning).

#### Number of casual partners

Thirty-seven percent of men reported ten or more casual sexual partners in the last six months,

#### Circumcision status

A third (34%) of the men reported being uncircumcised.

#### STIs

The prevalence of anal warts diagnoses made between cohort study visits was 4% and the prevalence of anal gonorrhoea diagnoses made at study visits was <1%.

### Hazard ratios

Table 1 provides the hazard ratios for each factor considered in this study. A total of 53 HIV seroconversions were observed during the follow-up period of the cohort with an overall incidence rate of 0.78 per 100 person-years (95%CI:0.59-1.02). The risk factor analysis was performed on data associated with 47 HIV seroconverters for whom sexual behaviour data were available within 12 months of seroconversion.

#### UAI by reported HIV status of sexual partners

Compared to no UAI, UAI with any HIV-positive sexual partners was significantly associated with 16.1 times the risk of HIV seroconversion (95%CI:6.4-40.5); UAI with unknown status sexual partners 4.4 times the risk of HIV seroconversion (95%CI:1.8-11.2 and UAI with HIV-negative partners only, 2.2 times the risk of HIV seroconversion (95%CI:0.9-5.4) but this did not reach statistical significance.

#### UAI by sexual position

Compared to no UAI, receptive UAI with ejaculation was significantly associated with 4.7 times the risk of HIV seroconversion (95%CI:2.0-10.8) and receptive UAI with withdrawal 5.0 times the risk of HIV seroconversion (95%CI:1.9-12.9). There was no significant increased risk of HIV seroconversion associated with insertive UAI only (strategic positioning).

#### Number of casual partners

Compared with less than ten partners, reporting ten or more casual sexual partners in the last six months was associated with 2.1 times the risk of HIV seroconversion (95%CI:1.12-3.7).

#### Circumcision status

Compared to being circumcised, being uncircumcised was not significantly associated with HIV seroconversion (HR = 1.2, 95%CI:0.7-2.2).

#### STIs

Anal gonorrhoea was associated with 8.5 times the risk of HIV seroconversion (95%CI:2.6-28.0) and anal warts 5.2 times the risk of HIV seroconversion (95%CI:2.4-11.2).

### Population attributable risk

Tables 2 and 3 provide the PAR estimated for each factor included in multivariate model 1 and 2, respectively. In model 1, the risk factors selected for the population attributable risk analysis accounted for 91% (95%CI:81-97%) of HIV seroconversions. In model 2, the risk factors accounted for 94% (95%CI:82-98%) of HIV seroconversions.

**Table 2:** PAR of HIV seroconversion risk factors including UAI by HIV status of sexual partners

Risk factor	PAR (95% CI) Crude model	PAR (95% CI) Adjusted model	Average annual number of HIV cases	Average lifetime HIV costs **(range)**^**(1) **^**AUD$million, 2010 dollars**		
				**1% discounting**	**3% discounting**	**5% discounting**

**All risk factors**	-	0.91 (0.81,0.97)	637	434 (403-471)	274 (249-304)	180 (160-205)

**UAI by HIV status of sexual partners**^(2)^	0.85 (0.75,0.94)	0.77 (0.66,0.89)	539	367 (341-399)	232 (211-257)	152 (136-174)

UAI with HIV-negative only (serosorting)	0.15 (0.11,0.22)	0.10 (0.07,0.19)	70	48 (44-52)	30 (27-33)	20 (18-23)

UAI with some unknown HIV status	0.34 (0.27,0.43)	0.33 (0.26,0.42)	231	157 (146-171)	99 (90-110)	65 (58-74)

UAI with any HIV-positive	0.36 (0.26,0.45)	0.34 (0.24,0.44)	238	162 (151-176)	102 (93-114)	67 (60-77)

**Circumcision status**^(3)^						

Uncircumcision	0.08 (0.04,0.16)	0.07 (0.03,0.14)	49	33 (31-36)	21 (19-23)	14 (12-16)

**Number of sexual partners**						

10+ casual sexual partners in the last 6 m	0.26 (0.18,0.36)	0.19 (0.09,0.34)	133	91 (84-98)	57 (52-64)	38 (33-43)

**STIs**^(4)^						

Anal warts between study visits	0.14 (0.10,0.18)	0.13 (0.09,0.19)	91	62 (58-67)	39 (36-43)	26 (23-29)

Anal gonorrhoea at study visit	0.06 (0.04,0.07)	0.02 (0.01,0.03)	14	10 (9-10)	6 (5-7)	4 (4-5)

CI = Confidence interval, PAR = population attributable risk, UAI = Unprotected anal intercourse, m = months

1. The average healthcare costs incurred from HIV seroconversions associated with specific risk factors were calculated by multiplying the PAR percent for each specific risk factor, by the 700 HIV infections associated with male homosexual exposure, by the average healthcare costs per HIV-infected person. We performed sensitivity analyses by rate of discounting and according to the bounds in delays in durations of time between infection and diagnoses and commencement of antiretroviral treatment but calculations were based on the best estimated PAR of the risk factors and not the 95% CI of the PAR.

2. Adapted from Jin et al [8]

3. Adapted from Templeton et al [9]

4. Adapted from Jin et al [21]

**Table 3:** PAR of risk factors including UAI by sexual position

Risk factor	PAR (95% CI) Crude model	PAR (95% CI) Adjusted model	Average annual number of HIV cases	Average lifetime HIV costs **(range)**^**(1) **^**AUD$million, 2010 dollars**		
				**1% discounting**	**3% discounting**	**5% discounting**

**All risk factors**		0.94 (0.82,0.98)	658	448 (416-487)	283 (257-314)	186 (165-212)

**UAI by sexual position**^(2)^	0.78 (0.69,0.89)	0.73 (0.66,0.85)	511	348 (323-378)	220 (200-244)	144 (128-165)

Insertive UAI only (strategic positioning)	0.12 (0.07,0.19)	0.04 (0.02,0.10)	28	19 (18-21)	12 (11-13)	8 (7-9)

Receptive UAI	0.66 (0.54,0.76)	0.69 (0.58,0.78)	483	329 (305-357)	208 (189-231)	136 (121-156)

Receptive with withdrawal	0.21 (0.16,0.28)	0.28 (0.20,0.37)	196	134 (124-145)	84 (77-94)	55 (49-63)

Receptive with ejaculation	0.45 (0.35,0.55)	0.41 (0.32,0.51)	287	196 (182-212)	123 (112-137)	81 (72-92)

**Circumcision status**^(3)^						

Uncircumcision	0.08 (0.04,0.16)	0.07 (0.03,0.15)	49	33 (31-36)	21 (19-23)	14 (12-16)

**Number of sexual partners**						

10+ casual sexual partners in the last 6 m	0.26 (0.18,0.36)	0.25 (0.14,0.42)	175	119 (111-129)	75 (68-84)	49 (44-56)

**STIs**^(4)^						

Anal warts between study visits	0.14 (0.10,0.18)	0.12 (0.08,0.18)	84	57 (53-62)	36 (33-40)	24 (21-27)

Anal gonorrhoea at study visit	0.06 (0.04,0.07)	0.02 (0.01,0.03)	14	10 (9-10)	6 (5-7)	4 (4-5)

CI = Confidence interval, PAR = population attributable risk, UAI = Unprotected anal intercourse, m = months

1. The average healthcare costs incurred from HIV seroconversions associated with specific risk factors were calculated by multiplying the PAR percent for each specific risk factor, by the 700 HIV infections associated with male homosexual exposure, by the average healthcare costs per HIV-infected person. We performed sensitivity analyses by rate of discounting and according to the bounds in delays in durations of time between infection and diagnoses and commencement of antiretroviral treatment but calculations were based on the best estimated PAR of the risk factors and not the 95% CI of the PAR.

2. Adapted from Jin et al [8]

3. Adapted from Templeton et al [9]

4. Adapted from Jin et al [21]

#### UAI by reported HIV status of sexual partners

In model 1, UAI with any HIV-positive partner accounted for 34% (95%CI:24-44%) of HIV seroconversions, UAI with some unknown HIV status men accounted for 33% (95%CI:26-42%) of the HIV seroconversions and UAI with HIV-negative partners accounted for 10% of the HIV seroconversions (95%CI:7-19%).

#### UAI by sexual position

In model 2, receptive UAI with ejaculation accounted for 41% (95%CI:32-51%) of HIV seroconversions, receptive UAI with withdrawal accounted for 28% (95%CI:20-37%) of HIV seroconversions and insertive UAI only (strategic positioning) 4% (95%CI:0.2-10%) of HIV seroconversions.

#### Number of casual partners

Ten or more casual sexual partners in the past six months accounted for 19% (95%CI:9-34%) of HIV seroconversions in model 1 and 25% (95%CI:14-42%) in model 2.

#### Circumcision status

Being uncircumcised accounted for 7.0% of HIV seroconversions in model 1 and 2.

#### STIs

A study visit diagnosis of anal gonorrhoea was associated with 2% of HIV seroconversions in model 1 and 2. An interval diagnosis of anal warts was associated with 13% of HIV seroconversions (95%CI:9-19%) in model 1 and 12% (95%CI:8-18%) in model 2.

### HIV costs

Tables 2 and 3 also provide the average lifetime healthcare costs incurred from HIV seroconversions associated with specific risk factors in multivariate model 1 and 2, respectively.

#### UAI by reported HIV status of sexual partners

In model 1, UAI with any HIV-positive sexual partners led to an average lifetime incurred health care cost of $AUD102 million for the estimated 238 new infections each year associated with this risk behaviour (range: $AUD93 to $114 million) (3% discounting). UAI with some unknown HIV status sexual partners also led to a similar average lifetime incurred cost of $AUD99 million (range: $AUD99-110 million) and serosorting led to an average lifetime incurred cost of $AUD30 million (range: $AUD27 to $33 million) (Table 3).

#### UAI by sexual position

In model 2, receptive UAI with ejaculation led to an average lifetime incurred cost of $AUD123 million for the estimated 287 new infections each year associated with this risk behaviour (range: $AUD112 to $137 million), receptive UAI with withdrawal led to an average incurred cost of $AUD84 million (range: $AUD77 to $94 million), and insertive UAI only (strategic positioning) $AUD12 million (range: $AUD11 to $13 million).

#### Number of casual partners

Ten or more casual sexual partners in the past six months led to an average incurred health care cost of $AUD57 million for the estimated 133 new infections each year associated with this risk behaviour (range: $AUD52 to $64 million) in model 1. In model 2, the costs increased to $AUD75 million (range: $AUD68 to $84 million).

#### Circumcision status

Being uncircumcised led to an average incurred health care cost of $AUD21 million for the estimated 49 new infections each year associated with this risk (range: $AUD19 to $23 million) in model 1. In model 2 costs were similar: $AUD21 million (range: $AUD19 to $23 million).

#### STIs

In model 1 and 2, anal gonorrhoea led to an average incurred cost of $AUD6 million for the estimated 14 new infections each year associated with this risk factor, ranging from $AUD5 to $7. In model 1, anal warts led to an average incurred cost of $AUD39 million (range: $AUD36 to $43 million) and $AUD 36 million (range: $AUD 33 to 40 million) in model 2.

## Discussion

To our knowledge this study is the first attempt to investigate the PAR of HIV risk factors, and at the same time estimate the costs incurred as a result of these specific risk factors. The PAR represents an estimate of the proportion of infections eliminated, taking account of relationships with other variables. We found that UAI with any HIV-positive sexual partners was reported by 5% of men, the hazard ratio (HR) was 16.1 (95%CI:6.4-40.5), the PAR was 34% (95%CI:24-44%) and the average incurred healthcare costs was $102 million for the new infections each year associated with this risk factor. UAI with sexual partners of unknown HIV status was reported by 25% of men, the HR was 4.4 (95%CI:1.8-11.2), the PAR was 33% (95%CI:26-42%) and the incurred costs were $99 million. The prevalence of anal warts was 4%, the HR was 5.2 (95%CI:2.4-11.2), the PAR was 13% (95%CI:9-19%) and the incurred costs were $39 million for infections attributable to this risk factor each year.

The use of PAR allows us to estimate the numbers of infections in the population associated with specific behavioural practices and partner choice strategies. This information should assist in identifying how to optimally target health promotion activities. In practice, it is unlikely that the total elimination of a particular behaviour or partner choice strategy can be achieved by health promotion and some replacement behaviour may occur. Furthermore, strategies may have different costs per unit of impact. Therefore the findings of the PAR analysis should be viewed as one element in making decisions about health promotion strategies.

We found that although UAI with any HIV-positive sexual partner was a relatively low-prevalence behaviour, reported by 5% of men, it was associated with a 16-fold greater risk of HIV than avoidance of UAI altogether. As a result 34% of the population risk is attributable to this behaviour, a similar proportion as was attributable to the much more prevalent, but apparently less risky practice of UAI with some unknown HIV status partners. A previous study in the United States was able to determine that UAI with a partner of unknown HIV serostatus was associated with a PAR of 15% and UAI with a HIV-positive partner a PAR of 12%, however their PAR method did not adjust for inter-relatedness (and hence confounding) of the factors in the model [17].

Our analyses also demonstrated the population impacts of risk reduction strategies on HIV transmission. Serosorting was associated with a much lower risk than UAI with sexual partners of unknown or positive HIV status. However because the strategy was practiced by about a third of men it was associated with a PAR of 10%. Withdrawal was less commonly practiced (14% of men) but was associated with a five-fold significantly increased risk of HIV infection and thus a PAR of 28%. This suggests that even though these risk reduction strategies can be associated with some success in containing HIV at the population level they still account for a substantial number of HIV infections in the population. In HIV-negative men, the effectiveness of serosorting as a HIV prevention strategy will be compromised where there is uncertainty about the HIV status of the sexual partner. A study in Melbourne in 2008 demonstrated that of 639 gay men recruited from community venues 31% of the 61 men with HIV infection were unaware of their HIV-positive status. These men were highly sexually active, a third had never been tested for HIV before and another third self-reported their previous HIV test as being in the past six months [26].

We have developed a model that defines the PAR and costs incurred as a result of specific risk factors. If a health department is considering spending money on reducing risk, it is now possible to estimate the overall impact of the strategy on reducing HIV incidence. In our study, the PAR associated with gonorrhea as a HIV risk factor was 2% compared to 34% for UAI with any HIV-positive partners. This means that strategies that aim to eliminate gonorrhoea as a means of preventing HIV infection would need to cost 17-fold less to be more cost-effective (assuming that both strategies are equal in terms of the proportion of the target population that they are able to influence).

Our study has several limitations. First, participants in the HIM cohort were not selected randomly and findings may not be generalizable to the larger gay and homosexual community. Second, behavioural data were obtained by self-report and may be subject to recall and measurement bias. For example, in seven HIV cases in the HIM cohort, no UAI was reported. In some of these cases transmission may have occurred through oral sex [27,28] or due to transmission when a condom was used, but the risk of HIV attributable to these behaviours exclusively has been demonstrated to be very low [16]. Because there were seven HIV cases where no UAI was reported the overall PAR will never reach 100%. Third, the definitions of risk reduction behaviours were based on exclusive practice and can only be generalised to those who applied the strategy 100% of the time. Fourth, men do not engage in a single risk but cumulative risk events. Therefore a man's seroconversion may not just be due one single risk factor but multiple. Fourth, we did not separate UAI by partner type. In fact, the most common form of receptive UAI is with a regular partner who is known to be HIV-negative, and this does not convey a significantly increased risk of HIV infection [8]. Fifth, we only included the broad category of circumcision as a variable, whereas Templeton and colleagues found circumcision was associated with a significantly reduced risk of infection in the minority of men who expressed a preference for the insertive position during anal intercourse [9]. We were unable to restrict our PAR analysis to men with a preference for the insertive position as it limited the model to a small subset of the HIV serconversions. Finally, we only focused on the costs associated with HIV treatment and not other health care costs or societal costs such as time off work due to hospitalisations, or costs incurred for individuals diagnosed with HIV but not yet on HIV treatment.

## Conclusion

In summary, our results indicate that over a third of cases could be avoided by strategies focusing on eliminating UAI with a HIV-positive partner and UAI with any unknown HIV status partner. As anal warts are caused by HPV subtypes which are vaccine-preventable, a PAR of 13% of averted HIV infections should be factored into considering vaccination for young MSM. The model demonstrates that the cost benefits of interventions focused on specific risk factors may be substantial, particularly when the benefits of prevention of a combination of risk factors are considered together. Our results call for major efforts directed toward prevention in subsets of the population at highest risk for HIV.

## Competing interests

The authors declare that they have no competing interests.

## Authors' contributions

RG conceived the study, coordinated the study and drafted the manuscript. HW carried out the statistical analysis in collaboration with RG. DPW calculated the costs. GP, JJ, AEG, BD, DJT and DPW advised on behavioural and other risk factors to be included in the analysis, and the interpretation of the findings. JMK conceived the cost aspect of the study, participated in the design and helped to draft the manuscript. All authors read and approved the final manuscript.

## Pre-publication history

The pre-publication history for this paper can be accessed here:

http://www.biomedcentral.com/1471-2458/11/247/prepub
